# Metabolic mechanisms regulating distinct steps of the melanoma metastatic cascade

**DOI:** 10.1186/2049-3002-2-S1-P56

**Published:** 2014-05-28

**Authors:** Elena Piskounova, Michalis Agathocleous, Zeping Hu, Sean Morrison

**Affiliations:** 1 Children’s Research Institute, University of Texas Southwestern Medical Center, Dallas, TX, USA; 2 Howard Hughes Medical Institute, Chevy Chase, MD, USA

## Background

Metastasis is a complex multistep process: cancer cells have to detach from the bulk of the primary tumor, intravasate into the blood, survive anoikis and high shear forces in the blood, extravasate to a distal site, and finally proliferate to form metastatic nodules. It is unclear what molecular mechanisms enable some cancer cells to go through every step in the metastatic cascade. In particular, it is not known if the rare tumor cells that enter the circulation and grow at a distal site are metabolically different from the bulk of primary tumor cells, and if any such metabolic differences are important for metastasis.

## Results

Melanomas taken directly from patients were serially xeno-transplanted subcutaneously in severely immunocompromised mice. Distant metastasis in the mouse correlated strongly with distant metastasis in patients. Metabolomic profiling showed several metabolic differences between primary tumors and corresponding metastatic nodules. We are testing if these metabolic differences can control cellular signaling pathways, and if manipulation of metabolism can affect metastasis in vivo. Additionally, we are exploring differences in the mitochondrial biology of primary tumors and metastatic nodules. Finally, we are developing a method to detect the metabolic profile of rare circulating tumor cells using the same melanoma xenograft model in order to track the metabolic changes in melanoma cells at every step of the metastatic cascade.

## Conclusions

Identification of metabolic changes that occur during the metastatic cascade raises the possibility that metabolism is functionally important not only for primary tumor growth but also for tumor survival in the blood, colonization of distal organs, and formation of overt metastatic nodules. More detailed understanding of these metabolic mechanisms will allow us to identify new therapeutic targets to block metastasis.

